# HMM-ModE – Improved classification using profile hidden Markov models by optimising the discrimination threshold and modifying emission probabilities with negative training sequences

**DOI:** 10.1186/1471-2105-8-104

**Published:** 2007-03-27

**Authors:** Prashant K Srivastava, Dhwani K Desai, Soumyadeep Nandi, Andrew M Lynn

**Affiliations:** 1 School of Information Technology, Jawaharlal Nehru University, New Delhi, India

## Abstract

**Background:**

Profile Hidden Markov Models (HMM) are statistical representations of protein families derived from patterns of sequence conservation in multiple alignments and have been used in identifying remote homologues with considerable success. These conservation patterns arise from fold specific signals, shared across multiple families, and function specific signals unique to the families. The availability of sequences pre-classified according to their function permits the use of negative training sequences to improve the specificity of the HMM, both by optimizing the threshold cutoff and by modifying emission probabilities to minimize the influence of fold-specific signals. A protocol to generate family specific HMMs is described that first constructs a profile HMM from an alignment of the family's sequences and then uses this model to identify sequences belonging to other classes that score above the default threshold (false positives). Ten-fold cross validation is used to optimise the discrimination threshold score for the model. The advent of fast multiple alignment methods enables the use of the profile alignments to align the true and false positive sequences, and the resulting alignments are used to modify the emission probabilities in the original model.

**Results:**

The protocol, called HMM-ModE, was validated on a set of sequences belonging to six sub-families of the AGC family of kinases. These sequences have an average sequence similarity of 63% among the group though each sub-group has a different substrate specificity. The optimisation of discrimination threshold, by using negative sequences scored against the model improves specificity in test cases from an average of 21% to 98%. Further discrimination by the HMM after modifying model probabilities using negative training sequences is provided in a few cases, the average specificity rising to 99%. Similar improvements were obtained with a sample of G-Protein coupled receptors sub-classified with respect to their substrate specificity, though the average sequence identity across the sub-families is just 20.6%. The protocol is applied in a high-throughput classification exercise on protein kinases.

**Conclusion:**

The protocol has the potential to maximise the contributions of discriminating residues to classify proteins based on their molecular function, using pre-classified positive and negative sequence training data. The high specificity of the method, and increasing availability of pre-classified sequence data holds the potential for its application in sequence annotation.

## Background

Protein homology is used as the basis for studying its phylogeny and predicting its function. A preliminary step in annotation of protein function from its sequence, is to compare it against a database of functionally annotated sequences and infer function based on similar conservation patterns to known homologues. As databases of sequences with known functions are large, fast heuristic methods based on extending local alignments such as BLAST [1] and FASTA [2] are commonly employed for this task.

Improved sensitivity in detecting homologues is provided by profile-sequence comparison methods such as PSI-BLAST[3] – which uses position specific scoring matrices, and HMMER[4] which uses a profile Hidden Markov Model (HMM). A profile is developed from a multiple alignment and contains more information on the sequence family than a single sequence, providing a base for detecting homologs with discontinuous conservation patterns, and remote homologues.

Patterns of sequence conservation can arise from both phylogenetic and functional relationships between proteins [5]. Proteins perform a wide variety of functions, but share a comparatively small number of folds. The TIM-barrel fold, as an extreme example, includes oxidoreductases, lyases, hydrolases and isomerases, which are examples of divergent evolution of function within the fold [6]. These proteins, while within each class contain function-specific signals, share fold-specific signals across the functional groups. The development of profile-profile based methods, (e.g. HHSEARCH [7], COACH [8]) maximises the contribution of common signals between profiles, providing even greater sensitivity in detecting remote homologs, and have proven useful for fold classification. Profile HMM databases are now commonly used to assign a protein to a structural class: the Superfamily database[9] which maps profiles to SCOP[10] structures, and the Pfam database[11] – which is a database of protein families largely based on domains.

An important goal of sequence annotation is the ability to assign molecular function to a protein sequence. Phylogenomic inference attempts to annotate protein function in the context of its entire family, and though has improved accuracy and specificity, its universal applicability is hampered by the fact that it is a labor-intensive manual process that requires significant effort from dedicated scientists [12]. Sjolander and co-workers have used "sub-family HMMs", built from a multiple alignment of the protein family decomposed into functionally distinct sub-families, in classifying sequences with a very low error rate [13].

As sequences are increasingly being classified on the basis of their common function – e.g the Gene Ontology project [14] (see ref [12] for more examples), function specific profiles are important goals in the ability to annotate sequences. HMMs built from a functionally classified sub-family often pick up sequences belonging to other sub-families because of fold signals common to the family. Pre-classified data however, provides for the use of both positive and negative training sequences. Negative training sequences have been used before, both to modify emission probabilities [15] and transition probabilities [16]. Both methods employ the Viterbi algorithm to align negative training sequences to the model, and change its probabilities during the training stage. Hannehalli and Russell [17] have used positional entropy to assess the discerning value of an amino acid position in a multiple alignment, sub-classify sequences, and score sequences against HMMs to remove the influence of non-discriminating residues. Kernel based methods, notably the Support Vector Machine (SVM) have been applied to classify sequences both at the fold level[18] and at the sub-family level[19].

**Figure 1:**
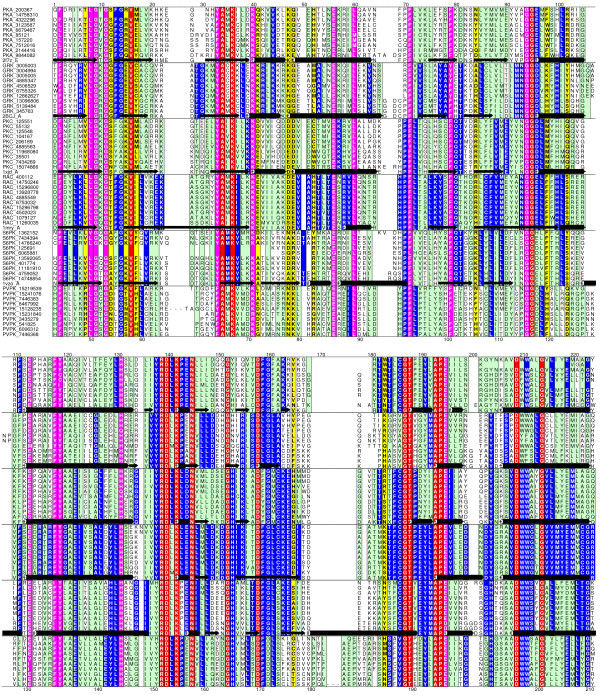
**A multiple alignment showing the common fold specific signals, along with the group specific sub-family function specific signals**. Alscript [36] figure showing a portion of the alignment of representatives of six protein kinase families discussed in the text. The alignment is coloured based on residue conservation: Red and pink – identical and conserved across all families – correspond to fold signals, and blue and green – identical and conserved within a family. Positions predicted to confer specificity for the family [35] are highlighted in yellow. Deleted regions are indicated by dashes (- - -). Numbers below the alignment correspond to the PDB structure 2f7z.

A multiple alignment of representative protein kinase sequences divided into sub-families is illustrative of the problem faced in using the HMM of a sub-family for classification (Figure 1). Easily apparent are the large number of columns which are conserved across all sub-families, representative of the fold signals. Amino acids selectively conserved in one sub-family are responsible for its specific function, and this information is used to discriminate sequences from the other sub-families. G-protein coupled receptors (GPCR) have also been classified hierarchically [20] and have been used to test the application of kernel based support vector machines as classifiers. This dataset provides another test for discriminating methods – they share a common fold but with limited sequence similarity across the family. As the HMM built from a family of sequences contains both common fold and function specific signals, the availability of a negative sequence data set allows the use of methods that optimise the discrimination threshold to separate sequences based on their function. Further separation, if necessary, maybe provided by modifying model parameters to minimize the influence of fold-specific signals and/or maximise the influence of specificity determining residues. These methods could be applied to classify proteins on the basis of their function in spite of their sharing a common fold.

We describe the use of cross-validation [21] to optimise the threshold to improve specificity for a particular sub-family profile HMM. From different measures of estimating classification accuracy, we choose the mode of the Matthews Correlation Coefficient (MCC) [22] distribution as the optimal threshold (referred henceforth in the text as HMM-t). In addition, improved specificity is imparted by using negative training sequences to modify model parameters – the emission and transition probabilities – to make the model more specific (referred henceforth as HMM-ModE). The method is applied in a high-throughput classification exercise to finely classify a sample derived from an earlier fold level data mining of protein kinases [23]. The sub-family profile HMM with default threshold will be referred to as HMM-d to differentiate from the profile HMM used with the optimal discriminating threshold, HMM-t.

## Results and Discussion

### Improved specificity is obtained by optimising the discrimination threshold

The HMM built from positive training sequences contains sub-family specific signals in addition to the common signals that arise from the family fold. The log-odds score, a measure of how much more probable the sequence is to be emitted by the given HMM than by the random null model, is routinely used in sequence profile comparison.

S=log⁡P(X1...Xn|M)P(X1...Xn|Null)
 MathType@MTEF@5@5@+=feaafiart1ev1aaatCvAUfKttLearuWrP9MDH5MBPbIqV92AaeXatLxBI9gBaebbnrfifHhDYfgasaacH8akY=wiFfYdH8Gipec8Eeeu0xXdbba9frFj0=OqFfea0dXdd9vqai=hGuQ8kuc9pgc9s8qqaq=dirpe0xb9q8qiLsFr0=vr0=vr0dc8meaabaqaciaacaGaaeqabaqabeGadaaakeaacqWGtbWucqGH9aqpcyGGSbaBcqGGVbWBcqGGNbWzdaWcaaqaaiabdcfaqjabcIcaOiabdIfaynaaBaaaleaacqaIXaqmaeqaaOGaeiOla4IaeiOla4IaeiOla4IaemiwaG1aaSbaaSqaaiabd6gaUbqabaGccqGG8baFcqWGnbqtcqGGPaqkaeaacqWGqbaucqGGOaakcqWGybawdaWgaaWcbaGaeGymaedabeaakiabc6caUiabc6caUiabc6caUiabdIfaynaaBaaaleaacqWGUbGBaeqaaOGaeiiFaWNaemOta4KaemyDauNaemiBaWMaemiBaWMaeiykaKcaaaaa@5200@

where *P*(X_1_,...X_n _| M) is the probability of the sequence X_1_...X_n _being emitted by the model M, and *P*(X_1_,...X_n _| Null) is the probability of the sequence being emitted by the null model.

All the sequences which obtain a positive score are considered to belong to family for which the model is built. The significance of this score, in HMMER, is calculated as an "E-value", assuming an extreme-value distribution whose parameters are either calculated during calibration, or from a conserved upper bound [24]. The use of the E-value is empirical, as the exact nature of the distribution of scores from global alignments is still unknown [24], though it has been shown that HMM score distributions are not an extreme-value distribution[25]. The Pfam database uses curated thresholds as an additional aid to the E-value: a "trusted" cutoff (TC1) – which is the lowest score of a true positive in the full alignment, a "noise" cut-off (NC1) which is the highest score for a sequence not included in the dataset, and a "gathering" threshold (GA1), which is the threshold that is actually set to collect the sequences in the Pfam Full alignment where TC1>GA1>NC1 [11]. These criteria cannot be uniformly applied to pre-classified positive and negative sequence data, as there maybe negative sequences with higher scores than positive sequences.

**Figure 2:**
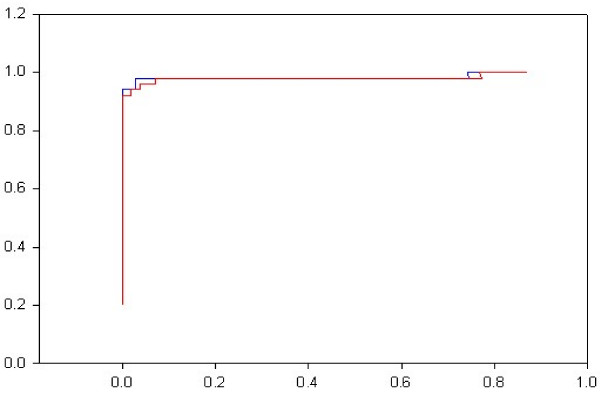
**A Receiver-Operator Characteristic curve (ROC) of HMM-d and HMM-ModE for the PVPK sub-family**. HMM-ModE – blue; HMM-d – red;

**Figure 3:**
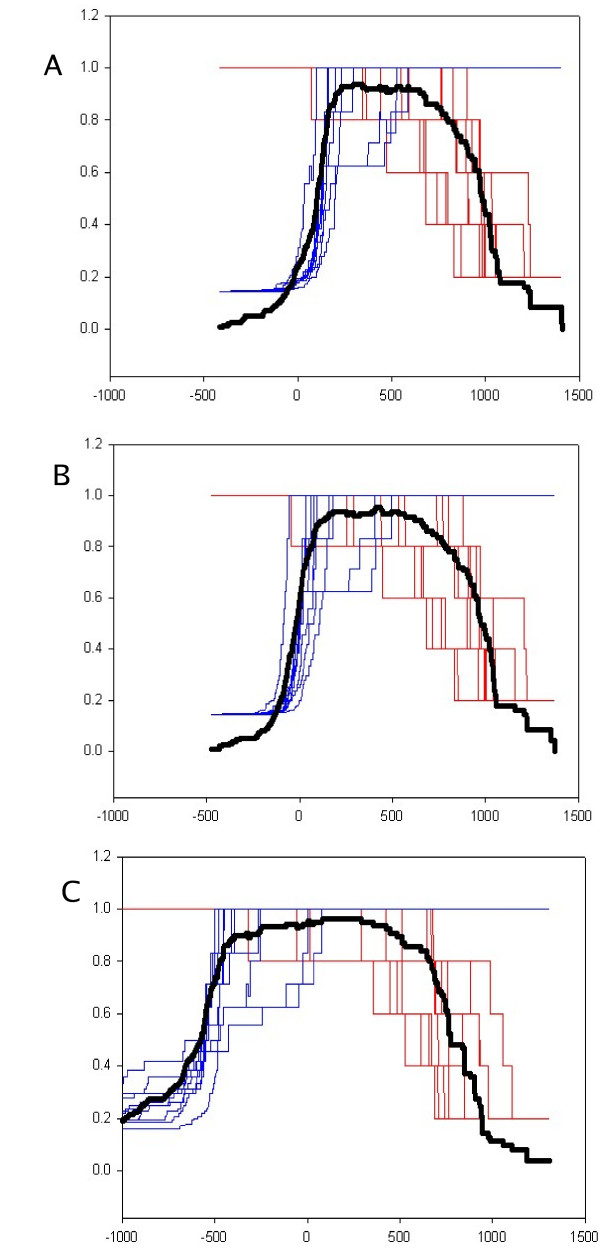
**Determination of optimal discrimination threshold**. The average MCC(bold black) distribution is overlayed on the sensitivity and specificity plots for each of 10-fold cross validation samples of the PVPK sub-family. Figures are plotted for the default profile HMM-d (top, A), HMM-ModE (center, B) and HMM-Sub(bottom, C).

For a given threshold score, a sequence from the positive set will be classified as a true positive(TP) or false negative(FN), and one from the negative set as a true negative(TN) or false positive(FP). Using these terms, sensitivity (TP/(TP+FN)) and specificity (TP/(TP+FP)) maybe used to measure the performance of a classifier. Receiver-Operator Characteristic curves (1-specificity v/s sensitivity) [22] show that the discriminating potential of the default HMM profile is inherently high and that the poor specificity of the HMM, generated from positive training sequences, results from the default threshold based on null probabilities (Figure 2). We use the Mathews correlation coefficient to indicate the optimal threshold. Normally used methods for testing the efficacy of discrimination in machine learning methods include cross-validation, where the sample is split into training and test data, and bootstrapping, where the data is randomly sampled multiple times [21]. N-fold cross-validation or jackknifing ranges from "sample-splitting" – where the sample is split equally into a test and training set, to "leave-one-out" – where the method is tested iteratively through the sample set, using as the test set a single sequence, and the remainder of the sample as a training set. In order to allow the method to be used in high-throughput analysis, we use 10-fold cross-validation, which lies between the accuracy of bootstrapping, and the speed of sample-splitting. We use the mode of the average MCC distribution as the optimal discrimination threshold. (Figure 3A).

### Further improvement in discrimination is provided by using negative sequences to train the HMM

Increased discrimination is provided by modifying emission and transition probabilities in the model by incorporating probabilities derived from negative training sequences directly into the model. Earlier work that used HMM with discriminative training modified model emission probabilities by iteratively aligning negative sequences to the model [15]. This method uses the capabilities of the HMM to both generate the multiple alignment and train the model with positive and negative sequences, a feature which is not available with HMMER, which uses a null model containing position independent probabilities derived from background frequencies of the amino acids. Moreover, multiple alignments generated from HMMs are not as accurate as methods employing scoring matrices – the profiles from Pfam are often hand-edited, and our use of *hmmalign *[24] to align negative training data does not generate alignments of the quality as specialized multiple alignment programs working from sequences (data not shown). The advent of fast and accurate multiple alignment programs such as MUSCLE [26] permits the generation of the model parameters by using profile-profile alignments of the positive and negative samples. Analysis of these profiles allows the easy identification of alignment positions capable of increased discrimination, and the modification of model parameters to implement them. Discriminating alignment positions can be identified using relative entropy(RE_*i*_) between the probability distributions of the positive (*p*) and negative (*q*) sets for a position *i *[27,28].

REi=∑pi,xlog⁡pi,xqi,x
 MathType@MTEF@5@5@+=feaafiart1ev1aaatCvAUfKttLearuWrP9MDH5MBPbIqV92AaeXatLxBI9gBaebbnrfifHhDYfgasaacH8akY=wiFfYdH8Gipec8Eeeu0xXdbba9frFj0=OqFfea0dXdd9vqai=hGuQ8kuc9pgc9s8qqaq=dirpe0xb9q8qiLsFr0=vr0=vr0dc8meaabaqaciaacaGaaeqabaqabeGadaaakeaacqqGsbGucqqGfbqrdaWgaaWcbaGaemyAaKgabeaakiabg2da9maaqaeabaGaemiCaa3aaSbaaSqaaiabdMgaPjabcYcaSiabdIha4bqabaGccyGGSbaBcqGGVbWBcqGGNbWzdaWcaaqaaiabdchaWnaaBaaaleaacqWGPbqAcqGGSaalcqWG4baEaeqaaaGcbaGaemyCae3aaSbaaSqaaiabdMgaPjabcYcaSiabdIha4bqabaaaaaqabeqaniabggHiLdaaaa@47A7@

where *p*_*i*,*x *_and *q*_*i*,*x *_are the probabilities of the amino acid *x *at a position *i *in the positive and negative sets respectively.

In order to use a model independent method of selecting discriminating alignment positions, Z-scores based on the distribution of cumulative relative entropies (CRE_*i*_) for the alignment may be used.

CREi=∑x=1...20pi,xlog⁡pi,xqi,x+∑x=1...20qi,xlog⁡qi,xpi,xZi=CREi−μσ
 MathType@MTEF@5@5@+=feaafiart1ev1aaatCvAUfKttLearuWrP9MDH5MBPbIqV92AaeXatLxBI9gBaebbnrfifHhDYfgasaacH8akY=wiFfYdH8Gipec8Eeeu0xXdbba9frFj0=OqFfea0dXdd9vqai=hGuQ8kuc9pgc9s8qqaq=dirpe0xb9q8qiLsFr0=vr0=vr0dc8meaabaqaciaacaGaaeqabaqabeGadaaakeaafaqaaeGabaaabaGaee4qamKaeeOuaiLaeeyrau0aaSbaaSqaaiabdMgaPbqabaGccqGH9aqpdaaeqbqaaiabdchaWnaaBaaaleaacqWGPbqAcqGGSaalcqWG4baEaeqaaOGagiiBaWMaei4Ba8Maei4zaC2aaSaaaeaacqWGWbaCdaWgaaWcbaGaemyAaKMaeiilaWIaemiEaGhabeaaaOqaaiabdghaXnaaBaaaleaacqWGPbqAcqGGSaalcqWG4baEaeqaaaaakiabgUcaRmaaqafabaGaemyCae3aaSbaaSqaaiabdMgaPjabcYcaSiabdIha4bqabaaabaGaemiEaGNaeyypa0JaeGymaeJaeiOla4IaeiOla4IaeiOla4IaeGOmaiJaeGimaadabeqdcqGHris5aOGagiiBaWMaei4Ba8Maei4zaC2aaSaaaeaacqWGXbqCdaWgaaWcbaGaemyAaKMaeiilaWIaemiEaGhabeaaaOqaaiabdchaWnaaBaaaleaacqWGPbqAcqGGSaalcqWG4baEaeqaaaaaaeaacqWG4baEcqGH9aqpcqaIXaqmcqGGUaGlcqGGUaGlcqGGUaGlcqaIYaGmcqaIWaamaeqaniabggHiLdaakeaacqWGAbGwdaWgaaWcbaGaemyAaKgabeaakiabg2da9maalaaabaGaem4qamKaemOuaiLaemyrau0aaSbaaSqaaiabdMgaPbqabaGccqGHsisliiGacqWF8oqBaeaacqWFdpWCaaaaaaaa@7D20@

where *μ *and *σ *are the mean and standard deviation of the CRE distribution.

The log-odds score is then given by

S=∑z>0log⁡pi,xqi,x
 MathType@MTEF@5@5@+=feaafiart1ev1aaatCvAUfKttLearuWrP9MDH5MBPbIqV92AaeXatLxBI9gBaebbnrfifHhDYfgasaacH8akY=wiFfYdH8Gipec8Eeeu0xXdbba9frFj0=OqFfea0dXdd9vqai=hGuQ8kuc9pgc9s8qqaq=dirpe0xb9q8qiLsFr0=vr0=vr0dc8meaabaqaciaacaGaaeqabaqabeGadaaakeaacqWGtbWucqGH9aqpdaaeqbqaaiGbcYgaSjabc+gaVjabcEgaNbWcbaGaemOEaONaeyOpa4JaeGimaadabeqdcqGHris5aOWaaSaaaeaacqWGWbaCdaWgaaWcbaGaemyAaKMaeiilaWIaemiEaGhabeaaaOqaaiabdghaXnaaBaaaleaacqWGPbqAcqGGSaalcqWG4baEaeqaaaaaaaa@434D@

High Z-scores (Z > 3) are associated with specificity determining positions [17], but although this method may work well on classifying sequences at the sub-family level that have been previously classified to the family level, it is insufficient to accurately mine a large database. The use of Z-scores to select variables (alignment positions) involved in maximal discrimination loses information that is shared between the positive and negative sequences, increasing the likelihood of an unrelated sequence that may contain the reduced pattern by chance. This is particularly likely in cases where specificity is conferred by only a few residues, or even a single position [29]. We propose a mixed score – that would discriminate the sequence belonging to the subfamily against a sequence containing the pattern by chance by incorporating the fold components of the profile, and against sequences from other subfamilies, by incorporating information related to the specificity determining residues identified using relative entropy.

REiNeg=∑x=1...20pi,xlog⁡pi,xqi,xREiNull=∑x=1...20pilog⁡pi,xPxNullS=∑i=1...n{∑x=1...20log⁡pi,xqi,x,REiNeg>REiNull∧qi,x>pxNull∑x=1...20log⁡pi,xPxNull,otherwise
 MathType@MTEF@5@5@+=feaafiart1ev1aaatCvAUfKttLearuWrP9MDH5MBPbIqV92AaeXatLxBI9gBaebbnrfifHhDYfgasaacH8akY=wiFfYdH8Gipec8Eeeu0xXdbba9frFj0=OqFfea0dXdd9vqai=hGuQ8kuc9pgc9s8qqaq=dirpe0xb9q8qiLsFr0=vr0=vr0dc8meaabaqaciaacaGaaeqabaqabeGadaaakeaafaqaaeWabaaabaGaeeOuaiLaeeyrau0aaSbaaSqaaiabdMgaPnaaBaaameaacqqGobGtcqqGLbqzcqqGNbWzaeqaaaWcbeaakiabg2da9maaqafabaGaemiCaa3aaSbaaSqaaiabdMgaPjabcYcaSiabdIha4bqabaGccyGGSbaBcqGGVbWBcqGGNbWzdaWcaaqaaiabdchaWnaaBaaaleaacqWGPbqAcqGGSaalcqWG4baEaeqaaaGcbaGaemyCae3aaSbaaSqaaiabdMgaPjabcYcaSiabdIha4bqabaaaaaqaaiabdIha4jabg2da9iabigdaXiabc6caUiabc6caUiabc6caUiabikdaYiabicdaWaqab0GaeyyeIuoaaOqaaiabbkfasjabbweafnaaBaaaleaacqWGPbqAdaWgaaadbaGaeeOta4KaeeyDauNaeeiBaWMaeeiBaWgabeaaaSqabaGccqGH9aqpdaaeqbqaaiabdchaWnaaBaaaleaacqWGPbqAaeqaaOGagiiBaWMaei4Ba8Maei4zaC2aaSaaaeaacqWGWbaCdaWgaaWcbaGaemyAaKMaeiilaWIaemiEaGhabeaaaOqaaiabdcfaqnaaBaaaleaacqWG4baEdaWgaaadbaGaeeOta4KaeeyDauNaeeiBaWMaeeiBaWgabeaaaSqabaaaaaqaaiabdIha4jabg2da9iabigdaXiabc6caUiabc6caUiabc6caUiabikdaYiabicdaWaqab0GaeyyeIuoaaOqaaiabdofatjabg2da9maaqafabaWaaiqabeaafaqaaeGacaaabaWaaabuaeaacyGGSbaBcqGGVbWBcqGGNbWzdaWcaaqaaiabdchaWnaaBaaaleaacqWGPbqAcqGGSaalcqWG4baEaeqaaaGcbaGaemyCae3aaSbaaSqaaiabdMgaPjabcYcaSiabdIha4bqabaaaaaqaaiabdIha4jabg2da9iabigdaXiabc6caUiabc6caUiabc6caUiabikdaYiabicdaWaqab0GaeyyeIuoakiabcYcaSaqaaiabbkfasjabbweafnaaBaaaleaacqWGPbqAdaWgaaadbaGaeeOta4KaeeyzauMaee4zaCgabeaaaSqabaGccqGH+aGpcqqGsbGucqqGfbqrdaWgaaWcbaGaemyAaK2aaSbaaWqaaiabb6eaojabbwha1jabbYgaSjabbYgaSbqabaaaleqaaOGaey4jIKTaemyCae3aaSbaaSqaaiabdMgaPjabcYcaSiabdIha4bqabaGccqGH+aGpcqWGWbaCdaWgaaWcbaGaemiEaG3aaSbaaWqaaiabb6eaojabbwha1jabbYgaSjabbYgaSbqabaaaleqaaaGcbaWaaabuaeaacyGGSbaBcqGGVbWBcqGGNbWzdaWcaaqaaiabdchaWnaaBaaaleaacqWGPbqAcqGGSaalcqWG4baEaeqaaaGcbaGaemiuaa1aaSbaaSqaaiabdIha4naaBaaameaacqqGobGtcqqG1bqDcqqGSbaBcqqGSbaBaeqaaaWcbeaaaaaabaGaemiEaGNaeyypa0JaeGymaeJaeiOla4IaeiOla4IaeiOla4IaeGOmaiJaeGimaadabeqdcqGHris5aOGaeiilaWcabaGaem4Ba8MaemiDaqNaemiAaGMaemyzauMaemOCaiNaem4DaCNaemyAaKMaem4CamNaemyzaugaaaGaay5EaaaaleaacqWGPbqAcqGH9aqpcqaIXaqmcqGGUaGlcqGGUaGlcqGGUaGlcqWGUbGBaeqaniabggHiLdaaaaaa@EFF9@

where *Px_Null_*is the null probability of amino acid *x*. Other terms are as defined earlier.

This effectively calls for a position dependent null model, that incorporates information from the negative training sequences. Though the Viterbi algorithm uses a log score in aligning a sequence to a profile to prevent underflow errors, this score is calculated from the model emission probabilities. In order to preserve the *plan7 *architecture used in HMMER, we use a heuristic method that modifies the model emission probabilities to implement this mixed model score.

The mixed model above still does not capture all information available from the false positive sequences. Consider the case where there is an conserved insert in the negative sequences that is absent in the positive sequences. As there is no equivalent emission probabilities in the positive profile (the matching columns in the positive profile HMM would be delete states), this information is lost. A trivial implementation of the log-odds score with known positive and negative sequences maybe made by scoring the sequence against the profiles generated from both positive sequences and negative sequences, and subtracting the negative profile score from the score of the sequence against the positive profile.

S=log⁡P(X1...Xn|MPos)P(X1...Xn|MNeg)=log⁡(P(X1...Xn|MPos)P(X1...Xn|Null)×P(X1...Xn|Null)P(X1...Xn|MNeg))=log⁡P(X1...Xn|MPos)P(X1...Xn|Null)−log⁡P(X1...Xn|MNeg)P(X1...Xn|Null)
 MathType@MTEF@5@5@+=feaafiart1ev1aaatCvAUfKttLearuWrP9MDH5MBPbIqV92AaeXatLxBI9gBaebbnrfifHhDYfgasaacH8akY=wiFfYdH8Gipec8Eeeu0xXdbba9frFj0=OqFfea0dXdd9vqai=hGuQ8kuc9pgc9s8qqaq=dirpe0xb9q8qiLsFr0=vr0=vr0dc8meaabaqaciaacaGaaeqabaqabeGadaaakeaafaqadeWabaaabaGaem4uamLaeyypa0JagiiBaWMaei4Ba8Maei4zaC2aaSaaaeaacqWGqbaucqGGOaakcqWGybawdaWgaaWcbaGaeGymaedabeaakiabc6caUiabc6caUiabc6caUiabdIfaynaaBaaaleaacqWGUbGBaeqaaOGaeiiFaWNaemyta00aaSbaaSqaaiabdcfaqjabd+gaVjabdohaZbqabaGccqGGPaqkaeaacqWGqbaucqGGOaakcqWGybawdaWgaaWcbaGaeGymaedabeaakiabc6caUiabc6caUiabc6caUiabdIfaynaaBaaaleaacqWGUbGBaeqaaOGaeiiFaWNaemyta00aaSbaaSqaaiabb6eaojabbwgaLjabbEgaNbqabaGccqGGPaqkaaaabaGaeyypa0JagiiBaWMaei4Ba8Maei4zaC2aaeWaaeaadaWcaaqaaiabdcfaqjabcIcaOiabdIfaynaaBaaaleaacqaIXaqmaeqaaOGaeiOla4IaeiOla4IaeiOla4IaemiwaG1aaSbaaSqaaiabd6gaUbqabaGccqGG8baFcqWGnbqtdaWgaaWcbaGaemiuaaLaem4Ba8Maem4CamhabeaakiabcMcaPaqaaiabdcfaqjabcIcaOiabdIfaynaaBaaaleaacqaIXaqmaeqaaOGaeiOla4IaeiOla4IaeiOla4IaemiwaG1aaSbaaSqaaiabd6gaUbqabaGccqGG8baFcqWGobGtcqWG1bqDcqWGSbaBcqWGSbaBcqGGPaqkaaGaey41aq7aaSaaaeaacqWGqbaucqGGOaakcqWGybawdaWgaaWcbaGaeGymaedabeaakiabc6caUiabc6caUiabc6caUiabdIfaynaaBaaaleaacqWGUbGBaeqaaOGaeiiFaWNaemOta4KaemyDauNaemiBaWMaemiBaWMaeiykaKcabaGaemiuaaLaeiikaGIaemiwaG1aaSbaaSqaaiabigdaXaqabaGccqGGUaGlcqGGUaGlcqGGUaGlcqWGybawdaWgaaWcbaGaemOBa4gabeaakiabcYha8jabd2eannaaBaaaleaacqqGobGtcqqGLbqzcqqGNbWzaeqaaOGaeiykaKcaaaGaayjkaiaawMcaaaqaaiabg2da9iGbcYgaSjabc+gaVjabcEgaNnaalaaabaGaemiuaaLaeiikaGIaemiwaG1aaSbaaSqaaiabigdaXaqabaGccqGGUaGlcqGGUaGlcqGGUaGlcqWGybawdaWgaaWcbaGaemOBa4gabeaakiabcYha8jabd2eannaaBaaaleaacqWGqbaucqWGVbWBcqWGZbWCaeqaaOGaeiykaKcabaGaemiuaaLaeiikaGIaemiwaG1aaSbaaSqaaiabigdaXaqabaGccqGGUaGlcqGGUaGlcqGGUaGlcqWGybawdaWgaaWcbaGaemOBa4gabeaakiabcYha8jabd6eaojabdwha1jabdYgaSjabdYgaSjabcMcaPaaacqGHsislcyGGSbaBcqGGVbWBcqGGNbWzdaWcaaqaaiabdcfaqjabcIcaOiabdIfaynaaBaaaleaacqaIXaqmaeqaaOGaeiOla4IaeiOla4IaeiOla4IaemiwaG1aaSbaaSqaaiabd6gaUbqabaGccqGG8baFcqWGnbqtdaWgaaWcbaGaemOta4KaemyzauMaem4zaCgabeaakiabcMcaPaqaaiabdcfaqjabcIcaOiabdIfaynaaBaaaleaacqaIXaqmaeqaaOGaeiOla4IaeiOla4IaeiOla4IaemiwaG1aaSbaaSqaaiabd6gaUbqabaGccqGG8baFcqWGobGtcqWG1bqDcqWGSbaBcqWGSbaBcqGGPaqkaaaaaaaa@F573@

This log-difference-of-odds-scores (henceforth referred to as HMM-Sub) would provide the maximum discrimination between the positive and negative datasets, but has some caveats discussed below. The comparative impact of these methods is shown in Figure 3 for a randomly selected dataset. It is apparent that the MCC distribution is successively broader and has a higher maximum with each method, corresponding to increased discrimination between the positive and negative datasets.

### Validation

Emission probabilities were modified as described in methods. Existing methods for modifying transition probabilities [16] from negative training data sampling [30] to improve the efficiency of HMMER were used without change. As negative training data is significantly larger in size than positive training data, the speed of implementation of HMM-ModE is improved by only selecting false positives from the negative training data, thus limiting its size to those sequences that significantly influence discrimination. The use of profile-profile alignments also permits easy calculation of resultant models, as the match states of columns of the positive and negative training data are aligned.

**Figure 4:**
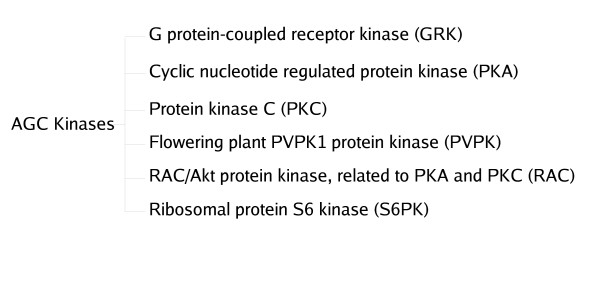
Six subfamilies of the AGC family of protein kinases

Protein Kinases provide the kind of challenge sub-family classification demands. Protein Kinases were first classified by Hanks[31] into distinct families that share basic structural and functional properties based on similarity in catalytic domain amino acid sequence, and more recently have been classified into 12 fold groups based on structural fold similarity. Each of the fold groups is further classified into families which can be distinguished from one another by representative HMMs [23]. Within the family, finer functional classification is often not possible due to the large proportion of shared fold signals. One such instance is the AGC family of Serine/Threonine Protein Kinases (Figure 4). The AGC family contains Protein Kinases such as cAMP-dependent Protein Kinase (PKA), Protein Kinase C, Protein Kinases related to PKA and PKC (RAC), G protein-coupled receptor kinase (GRK), ribosomal S6 PK, and the PVPK1 Protein Kinase homologs in plants [31]. The proteins all share a two-lobed structure and high level of sequence similarity, yet have different substrate specificity [32]. The results of the application of the above methods on this dataset is reported in Table 1. GRK has an insert relative to the other sequences, which is sufficient for HMMER with a suitable cut-off to improve its specificity. In all the other sub-families, the distribution of scores for positive and negative sequences overlap allowing an assessment of the discrimination capabilities of the methods described in this paper. In general, there is an increase in specificity using the HMM-ModE protocol, albeit with a reduction in sensitivity from the hmmer score with a default threshold. In the case of S6PK, the reduced sensitivity is due to three sequences in the dataset being shorter than the rest. As the log-odds-score increases with sequence length, this is an expected development, and must be used as a caveat for the general application of the method.

**Table 1:** Performance of HMM-d, HMM-t, HMM-ModE and HMM-Sub for the sub-family classification of the AGC family of kinases.

***Methods***	**HMM-d**				**HMM-T**				**HMM-ModE**				**HMM-Sub**			
***Sub-groups of AGC kinases***	**Sensitivity**		**Specificity**		**Sensitivity**		**Specificity**		**Sensitivity**		**Specificity**		**Sensitivity**		**Specificity**	
**GRK**	1	(0)	0.27	(0.03)	1	(0)	1	(0)	1	(0)	1	(0)	1	(0)	1	(0)
**PKA**	0.96	(0.1)	0.18	(0.02)	0.89	(0.13)	1	(0)	0.89	(0.13)	1	(0)	0.96	(0.07)	1	(0)
**PKC**	0.99	(0.05)	0.42	(0.1)	0.95	(0.08)	0.99	(0.2)	0.96	(0.08)	1	(0)	0.97	(0.06)	1	(0)
**PVPK**	1	(0)	0.17	(0.03)	0.94	(0.1)	0.93	(0.13)	0.94	(0.1)	0.96	(0.12)	0.96	(0.08)	1	(0)
**RAC**	1	(0)	0.09	(0.00)	0.9	(0.16)	1	(0)	0.93	(0.1)	1	(0)	0.97	(0.1)	1	(0)
**S6PK**	1	(0)	0.14	(0.01)	0.98	(0.08)	0.98	(0.06)	0.975	(0.08)	0.98	(0.06)	0.93	(0.24)	0.98	(0.06)

The values in parentheses indicate the standard deviation for all 10 samples in a 10-fold cross validation.

HMM-d – HMM profile used with default threshold

HMM-t – HMM profile used with optimised threshold

HMM-ModE – profile with modified emission probabilities

HMM-Sub – Log-difference-of-odds-score method

**Figure 5:**
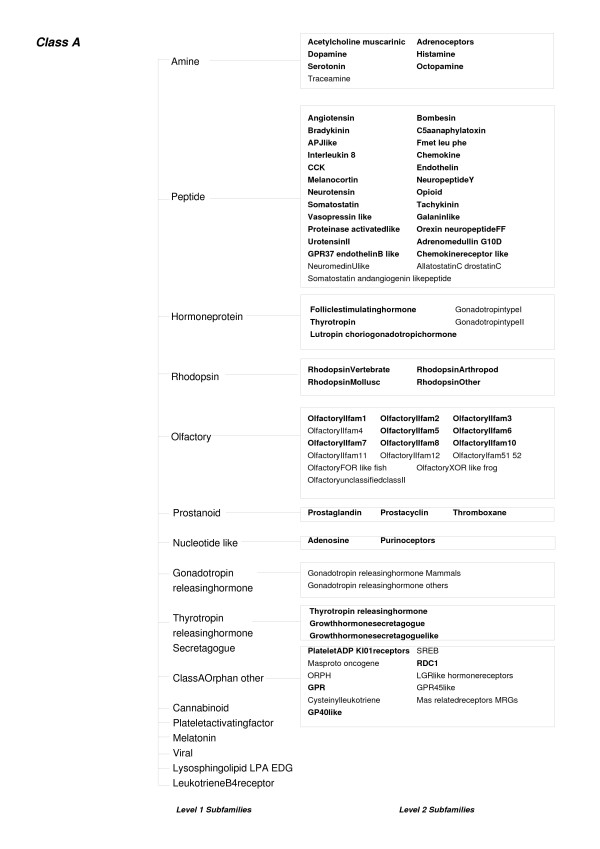
**An outline of some Level 1 and Level 2 subfamilies of the GPCR Class A proteins**. The level-2 sub-families used in this study are marked in bold.

**Figure 6:**
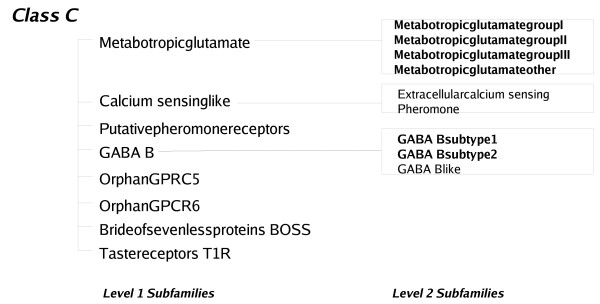
**An outline of some Level 1 and Level 2 subfamilies of the GPCR Class C proteins**. The level-2 sub-families used in this study are marked in bold.

G-Protein coupled receptors, which play a key role in cell-signaling network that control an array of physiological processes [33] have also been classified into sub-families on the basis of their substrate specificity[20]. These proteins are characterised by the conservation of seven transmembrane regions, the selection criteria being hydrophobic residues. Sequences from one sub-family often have higher sequence similarity with members of other families than within the sub-family. This dataset has also been the focus of the application of the SVM as a discriminator[19], and hence is interesting as it provides a comparison to the methods detailed above. Karchin *et al *have compared the relative performances of SVM, BLAST and HMMs for the classification of GPCR sub-families that bind to a specific ligand, defined by them as "level-2" sub-families (Figure 5 and 6). They calculate coverage (which is the percentage of True Positives selected before the first False Positive error) and the errors per sequence at the Minimal Error Point (MEP) as the parameters for evaluating the different methods, each of which could work best at different score thresholds. The former is indicative of the sensitivity of a discriminating method whereas the latter, since it is a total of both the False Positive as well as False Negative errors, indicates both sensitivity and specificity. These statistics are calculated by sweeping a threshold over the E-values combined from all the sub-families. The coverage values reported for SVM, BLAST and HMM are 65%, 13.3% and 5% respectively. Our HMM-d has a coverage of 13% which is comparable to the values reported for BLAST and HMM. The coverage of HMM-ModE (27%) is better than that of HMM-d. On the other hand, the 18% error at the MEP obtained for HMM-ModE is comparable to the 13.7% reported for SVM but lower than those reported for BLAST and HMM (25.5% and 30% respectively) or 21% obtained for HMM-d. However, we note that the average coverage and errors at MEP are calculated after combining and normalising results of different sub-families using the E-values. The results for HMM-ModE should be better than those observed using these parameters as sequence classification is based on the threshold score and not the E-value. A better comparison to the SVM results above, which also uses a discriminant score and not a significance value, would be to average sub-family values of these parameters. HMM-ModE then returns values of 96% coverage, and 6% error rate at MEP (Table 2).

**Table 2:** Coverage (percentage of True Positives identified before the first False Positive) and the average percentage of errors per sequence at the MEP of HMM-d and HMM-ModE for classification of Level-2 sub-families of Class A and Class C GPCR proteins

	**Coverage**				**Average errors per sequence at MEP**			
	**HMM-ModE**		**HMM-d**		**HMM-ModE**		**HMM-d**	
	**Set0**	**Set1**	**Set0**	**Set1**	**Set0**	**Set1**	**Set0**	**Set1**
**Acetylcholine_muscarinic**	1	1	1	0.92	0	0	0	0.08
**Adenosine**	1	1	1	1	0	0	0	0
**Adrenoceptors**	0.48	0.97	0.45	0.97	0.06	0.03	0.06	0.03
**Adrenomedullin_G10D**	1	1	1	1	0	0	0	0
**Angiotensin**	1	1	1	1	0	0	0	0
**APJlike**	1	1	1	1	0	0	0	0
**Bombesin**	1	1	1	1	0	0	0	0
**Bradykinin**	1	1	1	1	0	0	0	0
**C5aanaphylatoxin**	1	1	1	1	0	0	0	0
**CCK**	1	0.89	1	0.89	0	0.11	0	0.11
**Chemokinereceptor_like**	0.5	0.8	0.5	0.8	0.5	0.2	0.5	0.2
**Chemokine**	1	0.55	0.98	0.55	0	0.09	0.02	0.11
**Dopamine**	0.87	0.91	0.87	0.91	0.13	0.09	0.13	0.09
**Endothelin**	1	1	1	0.88	0.29	0	0.29	0.13
**Fmet_leu_phe**	1	1	1	1	0	0	0	0
**Folliclestimulatinghormone**	0.86	1	0.86	1	0.14	0	0.14	0
**GABA_Bsubtype1**	1	1	1	1	0	0	0	0
**GABA_Bsubtype2**	1	1	1	1	0	0	0	0
**Galaninlike**	1	1	1	1	0	0	0	0
**GP40like**	1	1	1	0.5	0	0	0	0.5
**GPR**	0.76	0.73	0.76	0.73	0.24	0.27	0.24	0.27
**Growthhormonesecretagoguelike**	1	1	1	1	0	0	0	0
**Growthhormonesecretagogue**	1	1	1	1	0	0	0	0
**Histamine**	1	1	1	1	0	0	0	0
**Interleukin_8**	1	1	1	1	0	0	0	0
**LeukotrieneB4receptorBLT1**	1	1	1	1	0	0	0	0
**Lutropin_choriogonadotropichormone**	1	1	1	1	0	0	0	0
**Melanocortin**	1	1	1	1	0	0	0	0
**MetabotropicglutamategroupIII**	1	1	1	1	0	0	0	0
**MetabotropicglutamategroupII**	1	1	1	1	0	0	0	0
**MetabotropicglutamategroupI**	1	1	1	1	0	0	0	0
**Metabotropicglutamateother**	1	1	1	1	1	1	1	1
**NeuropeptideY**	0.88	1	0.88	1	0.13	0	0.13	0
**Neurotensin**	1	1	1	1	0	0	0	0
**Octopamine**	0.29	1	0.29	1	0.71	0	0.71	0
**OlfactoryIIfam10**	1	1	1	1	0	0	0	0
**OlfactoryIIfam1**	0.86	0.80	0.86	0.80	0.14	0.10	0.14	0.10
**OlfactoryIIfam2**	1	1	0.88	1	0	0	0.13	0
**OlfactoryIIfam3**	1	1	1	1	0	0	0	0
**OlfactoryIIfam5**	1	0.71	1	0.71	0	0.29	0	0.29
**OlfactoryIIfam6**	0.83	1	1	1	0.17	0	0	0
**OlfactoryIIfam7**	1	1	1	0.86	0	0	0	0.14
**OlfactoryIIfam8**	1	1	1	1	0	0	0	0
**Opioid**	1	1	1	1	0	0	0	0
**Orexin_neuropeptideFF**	1	1	1	1	0	0	0	0
**PlateletADP_KI01receptors**	1	1	1	1	0	0	0	0
**Prostacyclin**	1	1	1	1	0	0	0	0
**Prostaglandin**	1	1	1	1	0	0	0	0
**Proteinase_activatedlike**	1	1	1	1	0	0	0	0
**Purinoceptors**	1	1	0.91	1	0	0	0.09	0
**RDC1**	1	1	1	1	0	0	0	0
**RhodopsinArthropod**	1	1	1	1	0	0	0	0
**RhodopsinMollusc**	0.6	1	0.6	1	0.4	0	0.4	0
**RhodopsinOther**	0.86	0.67	0.71	0.50	0.14	0.33	0.29	0.50
**RhodopsinVertebrate**	1	1	1	1	0	0	0	0
**Serotonin**	0.94	0.94	0.94	0.94	0.06	0.06	0.06	0.06
**Somatostatin**	1	1	1	1	0	0	0	0
**Tachykinin**	0.92	1	0.92	1	0.08	0	0.08	0
**Thromboxane**	1	1	1	1	0	0	0	0
**Thyrotropin_releasinghormone**	1	1	1	1	0	0	0	0
**Thyrotropin**	1	1	1	1	0	0	0	0
**UrotensinII**	1	1	1	1	0	0	0	0
**Vasopressin_like**	1	1	1	1	0	0	0	0
**Masproto_oncogene**	1	1	1	1	0	0	0	0
**GPR37_endothelinB_like**	1	1	1	1	0	1	0	1
**Average**	0.95	0.97	0.94	0.95	0.06	0.05	0.07	0.07
**Average(set0,set1)**	0.96		0.95		0.06		0.07	

The relatively poor results for Octopamine is due to the fact that there are only twelve sequences in the dataset, and they have higher sequence similarity with the serotonin sub-class than with each other (Table 2). Since our choice of threshold is optimised for specificity, there is a sharp fall in sensitivity. The HMM-ModE profile provides an improvement in the coverage values for 11 of the sub-families. Only in one case (OlfactoryII family6) is the coverage for HMM-ModE worse than HMM-d.

### Classification of kinases

**Figure 7:**
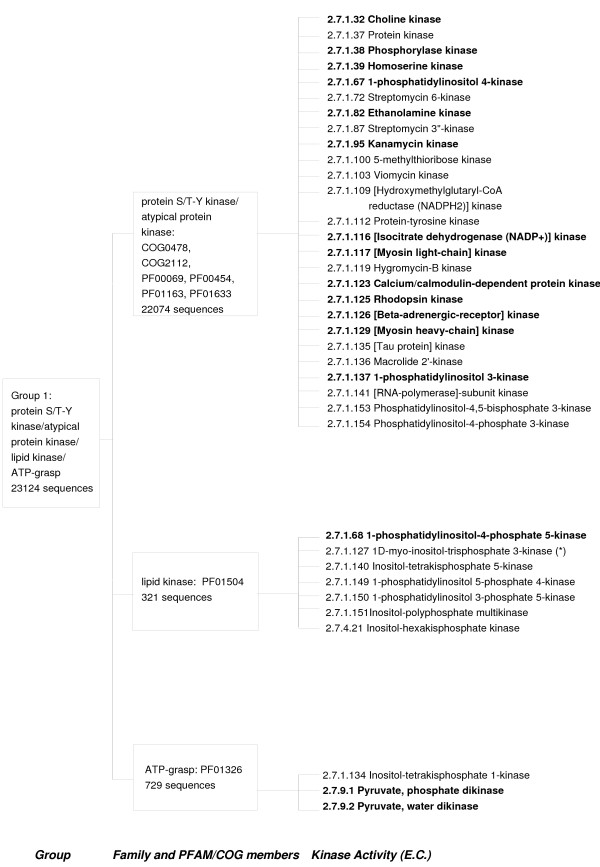
**An outline of the S/T-Y kinase/atypical kinase/lipid kinase/ATP-grasp Fold Group as categorized in [23]**. The EC numbers for which training sequences were available in the ENZYME database are marked in bold.

To test the above protocol in a high-throughput annotation case study, we applied the method to classify protein kinase sequences at a functional level. Protein Kinase sequences have been classified by Cheek *et al *[23] into fold groups on the basis of structural similarity and further into families of homologous sequences. Each family is made up of sub-families denoted by EC numbers. We constructed function-specific sub-family profiles using sequences from the ENZYME [34] database as a training set. The protein S/T – Y/atypical kinase/lipid kinase/ATP-grasp fold group contains enzymatic functions belonging to 36 different EC numbers, of which 19 EC numbers have 3 or more sequences available (Figure 7). We could populate the training set by mining databases for annotated sequences that fitted the description of the class, but used only specified sequences in this validation. Two generic activities, namely "protein kinase" and "protein tyrosine kinase" (EC numbers 2.7.1.37 and 2.7.1.112 respectively) were not included in the analysis.

The application of the method in high-throughput analysis is instructive. A careful perusal of the sequences classified showed that very few sequences with annotations outside of the sub-family were scored using HMM-ModE and HMM-t, commensurate with their expectation of high sensitivity and specificity. Rhodopsin and Beta-adrenergic receptor kinases are sub-families belonging to the G-protein-coupled receptor kinase 1 family which bind different substrates. The HMM-d profile for the Rhodopsin kinase sub-family (EC number 2.7.1.125) selected 18 sequences annotated as beta-adrenergic receptors from a database of 56,144 protein kinase sequences previously classified by Cheek *et al *[23]. The HMM-sub and HMM-d protocols each also classified 14 v-akt murine thymoma viral oncogene homologs as belonging to the rhodopsin kinase sub-family. HMM-ModE and HMM-t did not pick these false positives. Similarly, the HMM-d profile for the beta-adrenergic receptor kinase family (EC number 2.7.1.126) selected 5 rhodopsin kinase sequences whereas HMM-ModE, HMM-t and HMM-sub misclassified 3 rhodopsin kinases. Sequences mined with Pfam profiles as applied in Cheek *et al *[23] may not be specific at the functional level. For example, Pfam profile PF01633 is described as choline/ethanolamine kinase whereas choline kinase and ethanolamine kinase have different EC numbers signifying different substrate specificities (2.7.1.32 and 2.7.1.82 respectively). HMM-d, and HMM-t classisfied 45 and 31 sequences as ethanolamine kinases respectively. However, inspection of the classified sequences showed that 13 sequences annotated as choline kinases were also picked up by HMM-d but HMM-t only misclassified 3 choline kinase sequences. A similar trend was observed for the activities of phosphorylase kinase (EC number 2.7.1.38) and Calcium/Calmodulin dependent protein kinase (EC number 2.7.1.123). HMM-d, HMM-t, HMM-ModE and HMM-Sub misclassified 12, 1, 7 and 7 phosphorylase kinase sequences respectively as belonging to the Calcium/Calmodulin dependent protein kinase sub-family. It must be noted that in this case the number of correctly classified sequences for HMM-ModE was more than that of HMM-t(155 compared to 147). The HMM-d profile for phosphorylase kinase also selected 176 Calcium/Calmodulin dependent protein kinase sequences whereas the other methods did not pick up any sequence labeled as Calcium-Calmodulin Kinase.

The relatively high specificity of the HMM-ModE profiles and HMM-t provides a greater confidence with which to annotate unknown, hypothetical, putative or unnamed sequences. Table 3 shows the number of such sequences which have been annotated by our protocol.

**Table 3:** Application of HMM-d, HMM-t, HMM-ModE and HMM-Sub for function-specific classification of the S/T-Y kinase/atypical kinase/lipid kinase/ATP-grasp fold family

	**HMM-d**	**HMM-t**	**HMM-ModE**	**HMM-Sub**
2.7.1.100	19	16(0)	*	*
2.7.1.116	43	43(4)	*	*
2.7.1.117	103	103(8)	*	*
2.7.1.123	3392	529(120)	934(203)	3264
2.7.1.125	295	11(4)	11(4)	259
2.7.1.126	96	34(2)	34(2)	37
2.7.1.129	5	5(0)	*	*
2.7.1.137	135	135(29)	*	*
2.7.1.32	93	64(23)	*	*
2.7.1.38	2634	22(4)	22(4)	109
2.7.1.39	260	260(54)	*	*
2.7.1.67	57	57(15)	*	*
2.7.1.68	36	36(15)	*	*
2.7.1.82	45	31(10)	*	*
2.7.1.95	19	19(3)	*	*
2.7.9.1	171	169(55)	*	*
2.7.9.2	111	107(10)	*	*

The numbers in the parentheses for HMM-ModE and HMM-t are the total counts of sequences annotated as hypothetical, putative, unknown and unnamed which have been classified by the two protocols. A "*" in the HMM-ModE and HMM-Sub columns indicates that the number of false positive sequences picked up by the HMMER profile from the negative training data were not sufficient to build a false positive profile.

HMM-sub provides inconsistent results when used directly on a database of generic sequences. For protein families where the division of proteins into functional sub-types can be accomplished by phylogeny, this method would work well, as the specificity determining columns would then contain mutually exclusive amino acids in the different sub-families, and maximum discrimination would be provided by the application of this method. However at the level of classification we target the application of these methods, proteins usually have multiple features, not necessarily dependent on its molecular function that co-evolve. Examples include variations in sub-cellular location – membrane-bound or cytosolic, differing affinities for more than one substrate, or the interaction with other proteins that differ across paralogs. In addition, by effectively removing all features shared with the family, the method has a high chance of picking up false positives which may contain features unrelated to the molecular function.

Protein families whose members have convergently evolved is a case which will result in the failure of the methods described here. Though the catalytic activity of these proteins is the same, and the amino acids that confer this specificity would be similarly conserved in space, their arrangement in the sequence would be dependent on the scaffold of the protein fold. Although this case is not present in any of the case studies presented in this paper, we guard against this eventuality by first doing a phylogenetic clustering of the sequences from the subfamily. In the case of multiple folds, separate fold-based profiles maybe used. The clustering is also helpful in the case of very large training datasets, to allow sampling representative of the complete dataset to generate the multiple alignment.

## Conclusion

We have implemented a protocol to classify protein sequences based on profile HMMs. This protocol maximises the discrimination of the sequence belonging to the subfamily against a sequence containing the pattern by chance by incorporating the fold components of the profile, and against sequences from other subfamilies, by incorporating information related to the specificity determining residues identified using relative entropy. Although essentially implementing ideas suggested by Mamitsuka[15], Hannehalli and Russell[17], Wistrand and Sonnhammer[16], and Brown *et al *[13], this protocol is faster in training, as only negative sequences that are selected by the sub-family HMM as false positives, are used in modifying model parameters, and optimising the discrimination threshold. The use of HMMER for searching and scoring sequence databases remains unchanged except for our use of recent modifications to the model that aid improved discrimination. The hmmsearch E-value is no longer applicable as some of the model probabilities are modified to reflect information from negative training sequences, though the null probability used by the program to calculate the E-value still remains that of the original model as it is common across all states in the model.

The availability of accurately pre-classified protein sequences is an important starting point for classification based on function. Datasets classified on the basis of folds, such as the kinase set used in this work, additionally provide an opportunity for finer classification based on more specific function. Increased use of methods such as described in this manuscript, with a high prediction accuracy, will provide confidence in functional annotation of protein sequences which are generated from high-throughput genome sequence projects, a large proportion of which are of not experimentally characterised.

Given the above results, we recommend that if the coverage is 1 (i.e the highest false positive score is less than the lowest true positive score) the threshold as specified in Pfam may be used. If the coverage is less, then the threshold calculated using 10-fold cross-validation as described in this paper, is the optimal discriminating threshold for the given dataset, and can be used in place of the "gathering" threshold for sub-family classification. Further discrimination is possible if there are sufficient false positive sequences to build a profile.

## Methods

### Datasets used in the study

The sequences belonging to the 6 sub-families of the AGC protein kinase family, each with different ligand specificities, were kindly provided by the authors of [35]. These sequences included 66 sequences of cyclic nucleotide regulated Protein Kinases (PKA), 135 sequences of Diacylglycerol-activated/phospholipid-dependent protein kinase C (PKC), 23 sequences of RAC/Akt protein kinases, related to PKA and PKC, (RAC), 58 sequences of G protein-coupled receptor kinases (GRK), 40 sequences of protein kinases that phosphorylate the ribosomal protein S6 family (S6PK) and 48 sequences of the flowering plant protein kinase homolog family (PVPK1) (Figure 4).

Protein sequences belonging to level-2 sub-families of the Class A (Figure 5) and Class C GPCR (Figure 6) proteins were kindly provided by the authors of [19]. The sequences are a part of two mutually exclusive sets called set0 and set1 which are used in a two-fold cross validation exercise. These sets were manually inspected and were found to contain sequences whose accession numbers had either been deleted from the GenBank database or had been changed. The deleted entries were removed from the sequence sets and the changed entries were used to replace the old entries. Some other sequences which were found to have annotations of "probable", "putative" or "hypothetical" GPCRs were also removed. This resulted in a total of 542 sequences in set0 and 541 sequences in set1, respectively, which covered 65 level-2 subfamilies. The sequences for set0 were used as training sets and the HMM-d and HMM-ModE profiles were used to score the sequences in set1. The same procedure was repeated with set1 sequences as training sets and an average coverage and percentage errors per sequence at the Minimum Error Point (MEP) were calculated as in [19].

Protein Kinases previously classified upto the fold and family level by Cheek *et al *[23], were classified at the level of specific function as described by the Enzyme Commission (EC) numbers (Figure 7). The fold group of S/T-Y protein kinases/atypical kinases/lipid kinase/ATP-grasp sequences includes a total of 36 enzymatic activities of which 19 activities have 3 or more SWISSPROT sequences associated with them. Two enzymatic activities, "Protein kinase" and "Protein Tyrosine kinase" were ignored because they appear to be supersets of various other activities. The SWISSPROT sequences for each EC number were used to train HMMER and HMM-ModE profiles. These profiles were then used to classify 56,144 protein kinase sequences[23] obtained from the NCBI protein database.

### Identification of optimal cut-off score using cross-validation

The sequences were aligned using MUSCLE [26]. Hidden Markov Model (HMM) profiles were made from each of the multiple alignments by using program *hmmbuild *from the HMMER package version 2.3.2 [4]. The true positives and false positives were identified by scoring the sequences with the generated HMMER profiles using *hmmsearch*. In our study, we define True Positives (TP) as those protein sequence which perform the same specific function, that is, belong to the same specific subfamily with a score above the threshold value. A protein sequence from any of the other sub-families that has a score above the threshold, is considered as a False Positive (FP).

Ten-fold cross validation was used on each of the six sub-families for identifying an optimal discrimination threshold which would be used to classify the sequences with the HMMER profiles as well as with HMM-ModE. Each sub-family was divided into 10 samples of training and test sequences in the ratio 9:1 respectively, such that each sequence in a sub-group was a part of a test set at least once. Each training sample for each sub-family was further divided into 10 training and test sets using the same criteria as above. For each sub-family, the test set containing its own sequences was treated as a positive test while the corresponding test sets of the other groups were merged to form a negative test. The HMMER profiles and the HMM-ModE profiles generated from each of these training sets for each sample were used to score the corresponding positive and negative test sequences. The sensitivity, specificity and MCC (Matthews Correlation Coefficient)[22] distributions for 10 sets of each sample were obtained. An average MCC distribution for the 10 sets for a particular sample was plotted as a function of the scores. The optimal discrimination threshold was identified as the mid point corresponding to the mode valueof the average MCC distribution. The sequences in the each test sample for each sub-family were scored with HMMER and HMM-ModE profiles generated from the entire training sample using the optimal threshold score. The complete protocol is automated using PERL scripts along with the datasets are available from [37].

### Modifying the Emission probabilities of the HMMs (HMM-ModE)

The false positive (FP) set of sequences were aligned using MUSCLE. Profile alignment of the subfamily profile and the FP profile was performed using the *-profile *option of MUSCLE. HMMs for the aligned sub-family and FP alignments were built as described above. For nullifying the non-discriminating fold-specific emission probabilities the match emission probabilities of the true positive profile were modified with the emission probabilities of the FP profile using the equations described in the HMMER user's guide [24]. Specifically, the score in the HMM is modified using the condition

Score=integer(floor(0.5+(Intscale∗log⁡2(PPnull))))
 MathType@MTEF@5@5@+=feaafiart1ev1aaatCvAUfKttLearuWrP9MDH5MBPbIqV92AaeXatLxBI9gBaebbnrfifHhDYfgasaacH8akY=wiFfYdH8Gipec8Eeeu0xXdbba9frFj0=OqFfea0dXdd9vqai=hGuQ8kuc9pgc9s8qqaq=dirpe0xb9q8qiLsFr0=vr0=vr0dc8meaabaqaciaacaGaaeqabaqabeGadaaakeaacqWGtbWucqWGJbWycqWGVbWBcqWGYbGCcqWGLbqzcqGH9aqpieGacqWFPbqAcqWFUbGBcqWF0baDcqWGLbqzcqWGNbWzcqWGLbqzcqWGYbGCcqGGOaakcqWGMbGzcqWGSbaBcqWGVbWBcqWGVbWBcqWGYbGCcqGGOaakcqaIWaamcqGGUaGlcqaI1aqncqGHRaWkcqGGOaakcqWGjbqscqWGUbGBcqWG0baDcqWGZbWCcqWGJbWycqWGHbqycqWGSbaBcqWGLbqzcqGHxiIkcyGGSbaBcqGGVbWBcqGGNbWzdaWgaaWcbaGaeGOmaidabeaakiabcIcaOmaalaaabaGaemiuaafabaGaemiuaa1aaSbaaSqaaiabd6gaUjabdwha1jabdYgaSjabdYgaSbqabaaaaOGaeiykaKIaeiykaKIaeiykaKIaeiykaKcaaa@686B@

where *Intscale *= 1000

Probabilities can be derived from the scores using

P=Pnull∗2ScoreIntscale
 MathType@MTEF@5@5@+=feaafiart1ev1aaatCvAUfKttLearuWrP9MDH5MBPbIqV92AaeXatLxBI9gBaebbnrfifHhDYfgasaacH8akY=wiFfYdH8Gipec8Eeeu0xXdbba9frFj0=OqFfea0dXdd9vqai=hGuQ8kuc9pgc9s8qqaq=dirpe0xb9q8qiLsFr0=vr0=vr0dc8meaabaqaciaacaGaaeqabaqabeGadaaakeaacqWGqbaucqGH9aqpcqWGqbaudaWgaaWcbaGaemOBa4MaemyDauNaemiBaWMaemiBaWgabeaakiabgEHiQiabikdaYmaaCaaaleqabaWaaSaaaeaacqWGtbWucqWGJbWycqWGVbWBcqWGYbGCcqWGLbqzaeaacqWGjbqscqWGUbGBcqWG0baDcqWGZbWCcqWGJbWycqWGHbqycqWGSbaBcqWGLbqzaaaaaaaa@4945@

The modification steps and the generation of the new HMM in the HMMER format was accomplished using PERL scripts.

## Abbreviations used

HMM, Hidden Markov Model; HMM-d, Profile HMM used with default HMMER threshold; HMM-t, Profile HMM used with optimised discrimination threshold; HMM-ModE, Profile HMM with modified emission probabilities; HMM-Sub, log-difference-of-odds-scores; AGC, cAMP dependent protein kinase/protein kinase G/protein kinase C family; PKA, cAMP dependent protein kinase; PKC, Protein Kinase C; RAC, protein kinases related to PKA and PKC;GRK, G-Protein Coupled Receptor Kinase; S6PK, ribosomal S6 Protein Kinase; PVPK1, Flowering plant Protein Kinase; ROC, Receiver-Operator Characteristic; MCC, Matthew's Correlation Coefficient; MEP, Minimal Error Point.

## Authors' contributions

PKS and DKD wrote programs for the modification of emission probabilities, SN worked on the cross-validation and on modularising the final code, AML guided the implementation of the work. All the authors jointly wrote the manuscript.
